# The Role of Allogeneic Stem Cell Transplantation in Multiple Myeloma: A Systematic Review of the Literature

**DOI:** 10.7759/cureus.18334

**Published:** 2021-09-27

**Authors:** Arseni Khorochkov, Jose Prieto, Karan B Singh, Maduka C Nnadozie, Niki Shrestha, Jerry Lorren Dominic, Muhammad Abdal, Rose Anne M Abe, Anum Masroor, Lubna Mohammed

**Affiliations:** 1 Internal Medicine, California Institute of Behavioral Neurosciences & Psychology, Fairfield, USA; 2 Research, California Institute of Behavioral Neurosciences & Psychology, Fairfield, USA; 3 General Surgery, California Institute of Behavioral Neurosciences & Psychology, Fairfield, USA; 4 General Surgery, Stony Brook Medicine/Southampton Hospital, Southampton, USA; 5 General Surgery and Orthopaedic Surgery, Cornerstone Regional Hospital/South Texas Health System, Edinburg, USA; 6 General Surgery, LaSante Health Center, Brooklyn, USA; 7 Emergency Medicine, California Institute of Behavioral Neurosciences & Psychology, Fairfield, USA; 8 Psychiatry, California Institute of Behavioral Neurosciences & Psychology, Fairfield, USA; 9 Psychiatry, Psychiatric Care Associates, Englewood, USA; 10 Medicine, Khyber Medical College, Peshawar, PAK

**Keywords:** stem cell transplant for hematological malignancies, multiple myeloma, graft vs myeloma, allogenic bone marrow transplant, allogeneic stem cell transplant recipients

## Abstract

Multiple myeloma (MM) is an indolent B-cell malignancy, where treatment is aimed at preventing organ dysfunction from light chain accumulation (slowing disease progression) and inducing remission. Allogeneic stem cell transplant (allo-SCT), through graft versus myeloma (GVM) effects, has the potential to induce remission to a potentially curative-like state. In this systematic review, we aimed to understand this relationship to the risks and severity of disease in categorized patients and gain an updated comprehension of the future of allo-SCT in MM treatment. We conducted this review according to the Preferred Reporting Items for Systematic Reviews and Meta-Analyses (PRISMA) guidelines and searched the PubMed database to obtain the specified literature with both the use of keywords and Medical Subject Headings (MeSH). A total of 16 relevant articles were included for discussion after the quality appraisal was completed, as appropriate, by either the Cochrane tool or Newcastle-Ottawa checklist. Our review concludes that while allo-SCT may benefit high-risk patients, successful procedures may incorporate a tandem autologous hematopoietic stem cell transplant approach in combination with novel pharmacologic contributions for which there is an observed synergy in the modulation of the immunologic microenvironment. Furthermore, tailored patient selection by evaluating pre-transplant factors including high-risk cytogenetics, age, and pre-salvage International Staging System (ISS) can predict post-transplantation success including non-relapse mortality. Successive research should continue to revise and update treatment options as the evolving therapeutic drug regimens may change over the course of indolent disease.

## Introduction and background

Multiple myeloma (MM) affects five in every 100,000 people yearly [1] and makes up about 10% of all hematological cancers overall [1,2]. It is an indolent B-cell malignancy involving long-lived plasma cells, which remain in the bone marrow and produce antigen-specific immunoglobulin; however, malignant plasma cell clones produce an excess of light chains, which contribute to the pathology of the disease in addition to restraining the intended immune defense [3]. An asymptomatic precursor stage called smoldering MM (SMM) represents an intermediary between MM and indolent monoclonal gammopathy of unknown significance (MGUS) [4,5]. MGUS, affecting roughly 3% of people over the age of 50 years, converts to MM or a comparable malignancy yearly at about 1% [6].

Currently, the treatment of MM is aimed at preventing organ dysfunction from light chain accumulation (slowing disease progression) and inducing remission in far-progressed patients [4,7]. Although average survival has improved, post-diagnosis life expectancy remains around 7-10 years, making MM an incurable malignancy to this day [8].

Allogeneic hematopoietic stem cell transplantation (allo-SCT) has the potential to induce remission to a potentially curative-like state through graft versus myeloma (GVM) effects [9]. Sustained molecular remission accomplished by donor lymphocyte infusion (DLI) may evidence GVM effects; however, the standard of care for MM is a combination of autologous hematopoietic stem cell transplantation (auto-SCT) and high-dose melphalan, which itself as a conditioning agent is shown to reduce overall mortality [9-12]. Approved chemotherapeutic treatment for relapse and refractory cases of MM currently can involve a multidrug cocktail of panobinostat, bortezomib, and dexamethasone, which shows some clinical success [13-16].

Both allo-SCT and auto-SCT, despite procedural advances, may incur significant morbidity and mortality even with tailored patient selection [17,18]. Durable remissions have been shown in allo-SCT; however, the unacceptably high rates of treatment-related mortality are yet to be resolved; while auto-SCT exhibits less durable remission but a comparably lower rate of peri-transplantation mortality [2]. Although the use of allo-SCT remains controversial, to date, complete remission after allo-SCT is the most important prognostic factor for patients achieving long-term survival [19,20].

**Figure 1:**
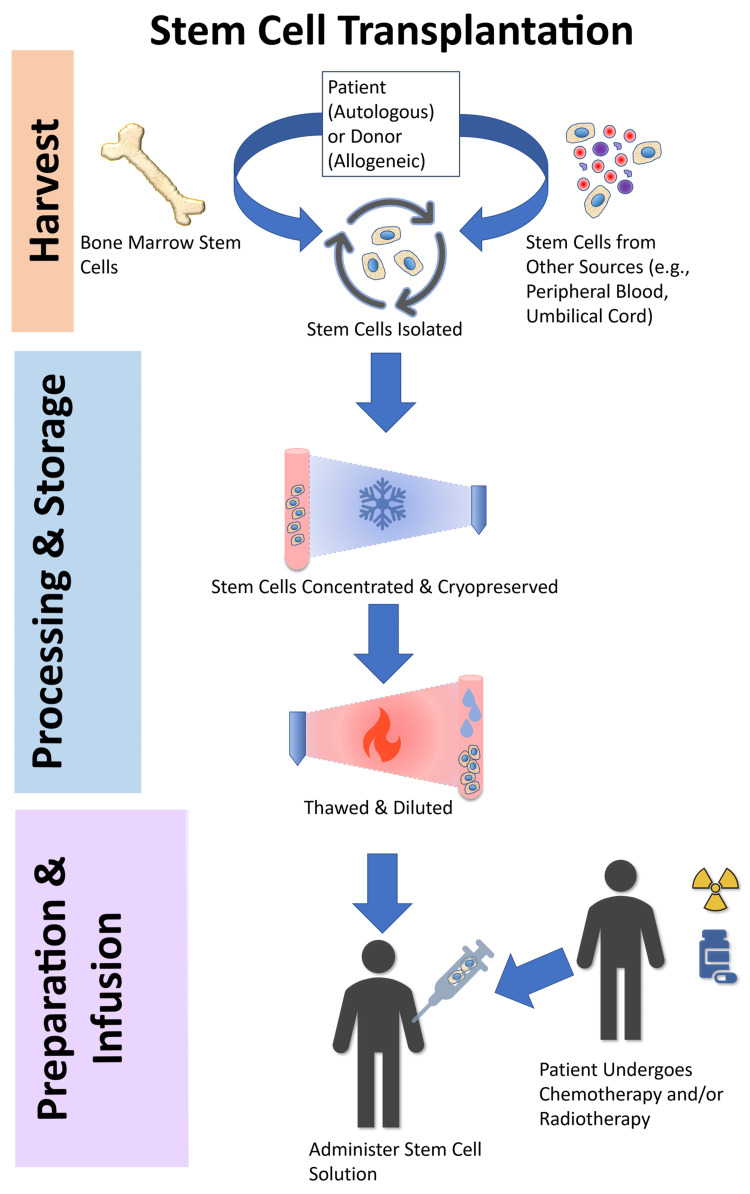
The general process of stem cell transplantation.

Currently, allo-SCT is considered a viable treatment option only in patients with severe disease [20]. While the risks and benefits must be carefully considered in any treatment modality, the potential reservoir of curative-like remission should be further evaluated, as allo-SCT is not the current standard of care. The high risks understood in allo-SCT treatment eliminate its potential as an option for treatment of non-responders in mild to moderate disease; however, the potentially curative success in some patient groups warrants further evaluation to elucidate mortality-reducing methods [20]. This systematic review explores the current literature on the use of allo-SCT in MM and evaluates when allo-SCT should be considered over other treatment options. Figure 1 demonstrates the general steps involved in stem cell extraction and transplantation [21].

## Review

Methods

Protocol

We performed a systematic review following the 2020 Preferred Reporting Items for Systematic Reviews and Meta-Analyses (PRISMA) guidelines [22].

Inclusion/Exclusion Criteria

We conducted a literature search to identify studies that assessed MM and stem cell transplantation (SCT), particularly allo-SCT. The criteria implored to gather relevant articles included (1) MM patients treated with allo-SCT and (2) any outcomes in MM patients considered for allo-SCT. We focused on chronic multiple myeloma patients without any age or gender discrimination. Articles that reported on MM patients undergoing auto-SCT without consideration for allo-SCT or MM exclusively treated with non-transplant therapy were ultimately out of the scope of this study and therefore were excluded.

Search Strategy

We systematically searched articles indexed in PubMed [23] and ScienceDirect [24] from January 1, 2016 to May 10, 2021. Generic keywords were used to search the database (“Bone Marrow Transplantation” OR “Stem Cell Transplant” AND “Multiple Myeloma” OR “Plasma Cell Myeloma”) and 119,458 studies were identified, and 3,904 remained after screening. We applied keywords and Medical Subject Headings (MeSH) terms individually and in combination with “adverse effects,” “immunology,” “methods,” “mortality,” “rehabilitation,” “therapeutic use,” and “therapy,” to identify relevant articles, which returned a total of 45,337 studies, 1,365 of which remained post-screening. Eligible studies were identified between January 1, 2016 and May 10, 2021 for full-texts available without cost and published in the English language. Only original research in the form of randomized control trials and observational studies were assessed in this review. Duplicate articles were removed in the screening process in EndNote. The results of the MeSH search strategy are detailed in Table 1.

**Table 1:** Database search results showing MeSH search strategy. MeSH, Medical Subject Headings.

MeSH strategy	Total articles	Inclusion/exclusion by automation
("Bone Marrow Transplantation/adverse effects"[Majr] OR "Bone Marrow Transplantation/immunology"[Majr] OR "Bone Marrow Transplantation/methods"[Majr] OR "Bone Marrow Transplantation/mortality"[Majr] OR "Bone Marrow Transplantation/rehabilitation"[Majr] OR "Bone Marrow Transplantation/therapeutic use"[Majr] OR "Bone Marrow Transplantation/therapy"[Majr])	11,017	70
("Multiple Myeloma/drug therapy"[Majr] OR "Multiple Myeloma/genetics"[Majr] OR "Multiple Myeloma/immunology"[Majr] OR "Multiple Myeloma/mortality"[Majr] OR "Multiple Myeloma/rehabilitation"[Majr] OR "Multiple Myeloma/surgery"[Majr] OR "Multiple Myeloma/therapy"[Majr])	17,160	648
Bone Marrow Transplant OR Stem Cell Transplant ("Bone Marrow Transplantation/adverse effects"[Majr] OR "Bone Marrow Transplantation/immunology"[Majr] OR "Bone Marrow Transplantation/methods"[Majr] OR "Bone Marrow Transplantation/mortality"[Majr] OR "Bone Marrow Transplantation/rehabilitation"[Majr] OR "Bone Marrow Transplantation/therapeutic use"[Majr] OR "Bone Marrow Transplantation/therapy"[Majr]) AND Multiple Myeloma OR Plasma Cell Myeloma ("Multiple Myeloma/drug therapy"[Majr] OR "Multiple Myeloma/genetics"[Majr] OR "Multiple Myeloma/immunology"[Majr] OR "Multiple Myeloma/mortality"[Majr] OR "Multiple Myeloma/rehabilitation"[Majr] OR "Multiple Myeloma/surgery"[Majr] OR "Multiple Myeloma/therapy"[Majr])	17,160	647

 Table 2 details keywords used in the search strategy.

**Table 2:** Database search results with regular keywords. PMC, PubMed Central.

Keywords	Database	Total articles	Inclusion/exclusion by automation
(((Bone Marrow Transplant) OR (Stem Cell Transplant)) AND (Multiple Myeloma)) OR (Plasma Cell Myeloma)	PubMed, PMC, Medline	55,919	1,576
	ScienceDirect	63,539	2,328

Data Extraction

Once the relevant articles were collected by authors AK and JP, the titles and abstracts, and full-texts were utilized in the final decision to include studies for discussion. Two independent researchers, AK and JP, convened on the decisions for scrutiny and accuracy.

Risk of Bias Assessment

The studies were assessed, as appropriate, to include those with moderate-to-high quality, with the following tools: (1) Newcastle-Ottawa checklist or (2) Cochrane risk-of-bias tool.

Results

Search Outcome

**Figure 2:**
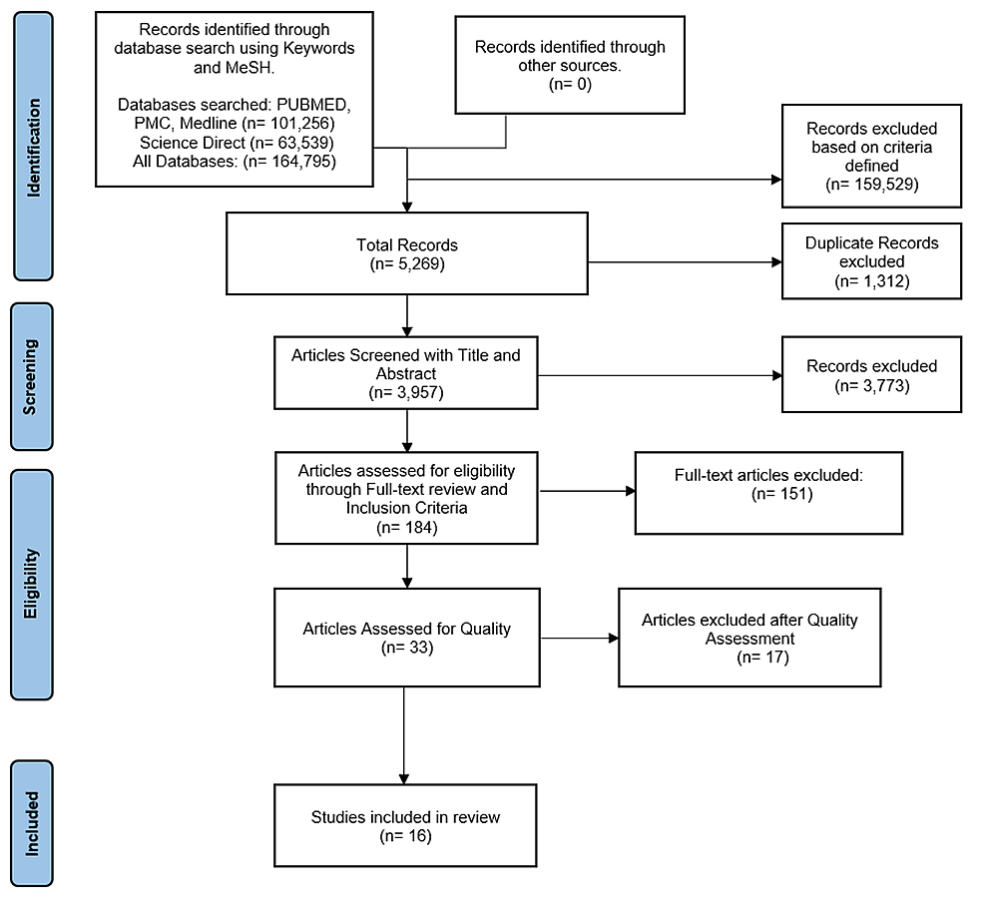
PRISMA flow diagram. PRISMA, Preferred Reporting Items for Systematic Reviews and Meta-Analyses; PMC, PubMed Central; MeSH, Medical Subject Headings; n, number of studies.

We collected relevant articles from PubMed, PubMed Central (PMC), Medline, and ScienceDirect databases. No other articles were identified using other sources. Our initial search yielded 164,795 articles without any restrictions. Once the inclusion/exclusion criteria were applied, 5,269 articles were attained: 3,904 identified with keywords and 1,365 using the MeSH strategy. After duplicates were removed (n = 1,312), 3,957 articles were screened by title and abstract for relevance, following which 3,773 non-relevant articles were excluded. Abstracts and full text of the 184 relevant articles that remained were thoroughly read, and 151 of them were excluded based on eligibility. A final quality assessment yielded 16 moderate-to-high quality observational and randomized control trials for inclusion in the review while the remaining 17 were further excluded. Figure 2 depicts the search process in the form of a PRISMA flow diagram.

A total of 16 peer-reviewed studies from 2016 to 2021 with free full texts that discussed the treatment of MM with allo-SCT were chosen for inclusion. They included both observational studies (n = 15) and randomized control trials (n = 1) with moderate-to-high quality based on the conducted bias assessment. The findings of these studies are summarized in Table 3 [25-40].

**Table 3:** Summary of articles included in the discussion. NK cells, natural killer cells; Haplo, haploidentical; SCT, stem cell transplantation; MM, multiple myeloma; CD, cluster of differentiation; CD34, adhesion molecule found on hematopoietic cells; allo, allogenic; HCT, hematopoietic cell transplantation; auto, autologous; RIT, radioimmunotherapy; RIC, reduced-intensity conditioning; HSCT, hematopoietic stem cell transplantation; PTCy, post-transplantation cyclophosphamide; BMT, bone marrow transplant; NRM, non-relapse mortality; GVHD, graft-versus-host disease; Treg cells, regulatory T cells; RGI-2001, a synthetic ligand that binds to CD1, activating, as well as expanding invariant natural killer cells; KIR2D, killer cell immunoglobulin-like receptors (KIRs) for HLA-C epitopes (found on NK cells); IPH2101, a novel KIR2D-specific antibody.

Author(s), year, [Reference]	Type of study	Patients	Purpose of study	Results
Van Elssen et al. (2021) [25]	Observational study	12	Observes the alloreactive effects of NK cells, in the context of haplo-SCT, and their ability to decrease the relapse of MM.	Haplo-SCT is a safe and viable transplant option as well as in later NK cell restoration. Patients did not show any significant improvement in progression-free survival.
Bryant et al. (2020) [26]	Observational study	73	Observes a subset of CD34+ relapsed refractory MM allo-HCT recipients and the effect of pre-allo-HCT factors on their outcomes.	Better disease control and survival can be seen in refractory patients with CD34+ allo-HCT. Worse outcomes with old age and more potent pretreatment.
Eisfeld et al. (2020) [27]	Observational study	90	Retrospective analysis of 90 allo-SCT recipients with MM, focusing on immunoparesis and post-transplant survival.	Select high-risk patients transplanted early in their disease course may improve long-term survival.
Holstein et al. (2020) [28]	Observational study	30	Compares disease progression in auto-SCT and auto-allo SCT.	Auto-SCT followed by allo-SCT in MM patients is safe and feasible. Further follow-up demonstrates long-term survival in some of the patients.
Giralt et al. (2020) [29]	Observational study	710	Compares auto-auto-HCT and auto-allo-HCT in standard and high-risk MM patients.	Early results of the study observed that allo-HCT patients did not show any changes in disease progression from auto-HCT. However, long-term survival was improved in the high-risk MM allo-HCT group.
Fasslrinner et al. (2020) [30]	Observational study	30	Retrospective analysis of 30 allo-HCT recipients with late-stage MM, who received RIT-RIC before allo-HCT transplantation.	RIT-RIC was a safe addition to pretreatment for allo-HCT in relapsed or refractory MM patients and had better outcomes than with RIC alone.
Gagelmann et al. (2019) [31]	Observational study	488	Compares single autologous, tandem autologous, and auto-allo SCT in high-risk/extramedullary disease MM patients.	Patients generally had worse outcomes with single autologous transplants; tandem autologous transplants may have a better prognosis; and auto-allo SCT results were encouraging but require further study.
López-Corral et al. (2019) [32]	Observational study	126	Observes effects of treatments in allo-SCT MM patients who underwent rescue therapies post relapse.	The study demonstrates that rescue therapies can be used in post-transplant patients safely and can improve response to drug therapy due to the newly formed immune system.
Sahebi et al. (2019) [33]	Observational study	96	Observe high-risk MM patients post-haplo-allo HCT.	Haplo-allo HCT in high-risk or relapsing MM patients can be a viable treatment option in conjunction with other post-transplant therapies but requires further exploration of the subject.
Kawamura et al. (2018) [34]	Observational study	65	Observes the effects of new therapies on MM patients who will be undergoing allo-HSCT.	Allo-HSCT is relatively safe to use in MM patients who have received prior therapies and has improved outcomes in the younger and more chemosensitive population.
Htut et al. (2018) [35]	Observational study	582	Compares overall survival of tandem autologous and auto-allo HCT in MM patients after relapse.	Post-relapse overall survival was better in MM patients with auto-allo HCT compared to tandem auto-auto HCT.
Giaccone et al. (2018) [36]	Randomized control trial	162	Compares survival in auto-SCT and allo-SCT after receiving new drugs and donor lymphocyte infusions.	Allo-SCT MM patients had better disease-free survival than auto-SCT.
Ghosh et al. (2017) [37]	Observational study	39	Observes the effects of post-transplantation PTCy in MM patients undergoing allo-BMT.	Patients had lower rates of NRM and GVHD with long-term remission in some patients.
Castagna et al. 2017) [38]	Observational study	30	Observes the survivability of haplo-HCT MM patients treated post-transplantation with cyclophosphamide prophylactically for GVHD.	Haplo-SCT with cyclophosphamide is a viable treatment option in the event when the matching donor is unavailable and in poor prognosis high-risk disease where novel pharmacotherapy was unsuccessful.
Chen et al. (2017) [39]	Observational study	29	Assess if increased Treg cells can reduce the risk of graft-versus-host disease in allo-HCT recipients with hematological malignancies.	RGI-2001 use in allo-HCT patients resulted in an increased Treg cell response in some and was generally safe in non-responders as well.
Carlsten et al. (2016) [40]	Observational study	9	Understanding the lack of clinical efficacy in KIR-ligand mismatched NK cells in reducing relapse of MM in allo-HCT recipients via administration of KIR2D-specific antibody, IPH2101.	Reduction of KIR2D on NK cells results in a corresponding decrease of NK cell function with only a small benefit to the treatment of MM.

Discussion

Our systematic review assessed 16 previously published studies to formulate a better understanding of the potentially curative results of allo-SCT in MM patients and its relationship to the risks and severity of disease in categorized patients. We intend to gain an updated comprehension of this relationship and the future of SCT in MM treatment. 

Combinative-Comparative Features in Allogeneic and Autologous SCT

Both allogeneic and autologous SCTs offer beneficial treatment options for eligible MM patients. The first-line treatment for newly diagnosed, transplant-eligible patients is high-dose pharmacotherapy with auto-hematopoietic cell transplant (HCT); however, conditions for treatment are stringent and concerns in previous studies involving auto-HCT involve high-risk cytogenetics (poor risk MM), including post-treatment time to relapse [25,31]. A small prospective study by Van Elssen et al. assessed if killer cell immunoglobulin-like receptor (KIR)-ligand mismatched haploidentical (haplo) bone marrow transplant (BMT) combined with post-transplant cyclophosphamide (PTCy) improves survival in poor-risk chemo-resistant MM, which, in this study, was not superior to conventional allo-HCT [25].

Nevertheless, a continued revision of therapy creates potential treatment options for patients in various categories. A study by Gagelmann et al. evaluated both clinical and cytogenetic data to assess patients with extramedullary disease undergoing auto-allo transplant, tandem autologous, or single-autologous transplant [31]. They found that under these circumstances, high-risk cytogenetics may impair outcomes after single autologous transplants; however, auto-allo transplant appeared to enhance survival but not necessarily outcomes [31]. Tandem autologous transplants may additionally surmount poor prognosis, particularly when utilized with the addition of bortezomib, lenalidomide, and dexamethasone when compared with standard therapy or single transplant [31]. Auto-allo transplant, in this study, also identified fewer occurrences of relapse compared with single or tandem autologous transplants, respectively [31]. Nevertheless, allogeneic transplant is considered the only potentially curative therapy and is proposed for younger, high-risk patients [31]. This study urges that the therapeutic role of auto-allo transplant needs a better definition for MM patients with high-risk disease as a first-line treatment, despite the limitations of small sample size [31]. Similarly, Holstein et al., Giralt et al., and Htut et al. have indicated that tandem auto-HCT followed by auto-allo HCT can have improved long-term survival in certain groups of patients [28,29,35]. Further insight is needed to determine the specific predictive parameters in which auto-allo HCT will be of most benefit [28].

A study by Giaccone et al. assessed drug-based treatment with combined autologous stem cell transplant and either (1) nonmyeloablative allo-SCT or (2) double auto-SCT [36]. Molecular remission in the allo-SCT group was notable, as the efficacy of the newer drug protocol assessed in combination with GVM benefitted the overall survival in this subset [36]. Induction with vincristine-Adriamycin-dexamethasone protocol in the allo-SCT group seems to have potentiated a synergism that promoted GVM [36]. Prognosis continues to be poor in high-risk patients with early relapse; however, post-relapse survival and overall long-term outcomes were significantly improved in the allo-SCT group compared with the auto-SCT group [36]. While allo-SCT itself may be a benefit to high-risk patients, successful procedures incorporate a combination with auto-SCT, and advances in drug induction, conditioning, and maintenance cannot be overstated [31,36]. The limitation of these studies in using small numbers and the long-term follow-up necessary to provide insight into the prognosis of MM with evolving availability of pharmacologic management requires further data.

Pharmacologic Contributions to SCT

The role of pharmacotherapy is exigent in allowing transplant intervention to take place. Nevertheless, new drug therapies are constantly evolving the landscape of treatment: the study by Van Elssen et al. describes haplo-BMT in MM may be a possible platform for future immunotherapeutic strategies utilizing the KIR-ligand mismatch; however, not necessarily with post-transplantation cyclophosphamide [25]. Immunoparesis was assessed by Eisfeld et al. in the context of post-transplant survival, in that it may aid as a gauge for post-allogeneic transplantation mortality [27]. Graft-versus-host disease (GVHD) and infection were the main causes of non-relapse mortality in this group, which speculatively may have resulted from myeloablative conditioning with busulfan and cyclophosphamide [27]. Similarly, the vincristine-Adriamycin-dexamethasone protocol in the Giaccone et al. study enhanced the non-relapse outcomes of allogeneic recipients [36].

Allo-HCT can modify the immuno-microclimate, which can contribute to the therapeutic response of drug regimens; however, many patients ultimately relapse after allo-HCT: the study by Lopez-Corral et al. investigated the safety and efficacy of relapsing MM patients post-allo-HCT [32]. They found that post-relapse overall survival was reduced in the absence of chronic GVHD and the majority of subjects responded well to rescue therapies involving immunomodulatory drugs and proteasome inhibitors to the degree of pre-transplantation period overall response [32]. Allo-HCT has also been established as a possible treatment option in patients who have undergone pretreatment with novel agents such as bortezomib or lenalidomide and can be particularly effective in younger, more chemosensitive patients [34]. Cyclophosphamide post-haplo allo-HCT can be an effective treatment option, having shown decreased rates of nonrelapse mortality as well as lower rates of GVHD and in poor prognostic high-risk disease where novel pharmacotherapy was unsuccessful [37,38]. A study by Sahebi et al. concluded that haplo allo-HCT compared with traditional donor-based transplants promotes an acceptable non-relapse mortality rate in MM patients without a matched donor; however, immune-based drug strategies enhance the anti-tumor effects and survival, including the use of immunomodulators, proteasome inhibitors, donor-derived Chimeric antigen receptor (CAR) T-cells, natural killer cell infusions, and bispecific killer cell engagers [33].

A phase IIa clinical trial by Chen et al. tested the concept that increasing regulatory T cells (Treg) may mitigate the risk of GVHD post-allo-HCT using RGI-2001, a synthetic derivative of a CD1 ligand that perpetuates invariant natural killer cells [39]. Although there was no control group to make a direct comparison, the medication was safe to use and can be given in conjunction with sirolimus to increase the Treg cell response, thereby further decreasing the risk of GVHD [39]. While the mechanism of action is not fully understood, it is speculated that the increase in Treg cells by RGI-2001 is via activation of invariant natural killer T (iNKT) cells [39]. Patients with greater than 9% CD4+ Treg cells have a greatly reduced risk of acute GVHD, and as a result, this could contribute to the prolonged overall survival of MM patients post-transplant [27,39].

However, not all theorized therapeutics are clinically effective, as evaluated by Carlsten et al. in the premature termination of a study on SMM involving IPH2101 hypothesized to induce KIR-ligand mismatched tumor killing through natural killer cells and could be shown in vitro but was limited in vivo by antibody-induced hypo-responsiveness [40]. They conclude that anti-KIR antibody therapy in SMM requires further study to determine if the combination of other agents could render this target useful for therapy [40]. Particularly, in allo-SCT, the synergistic effects are of interest to designing drug regimens that work with the host to achieve GVM effects. Further investigation must be done to understand this process and continue to develop and revise existing treatment regimens. Despite the wealth of treatment options available, the intrinsic qualities of the patient are considered when devising treatment plans. 

Peri-Transplant Factors Affecting Patient Outcome

While MM is considered largely incurable, patients who develop resistance to therapeutics may have hope for a curable-like state with allo-HCT [26]. However, eligibility and projected success depend heavily on pre-allo-HCT evaluation [26]. A study by Bryant et al. evaluated a cohort of CD34+ allo-HCT recipients with relapsed refractory MM and their pre-allo-HCT variables [26]. An important adverse pre-allo HCT variable was the pre-salvage stage II-III International Staging System (ISS), which resulted in relapse and poorer survival [25]. However, radioimmunotherapy (RIT) combined with reduced-intensity conditioning (RIC) in patients who responded to pre-salvage therapy before allo-HCT saw a benefit in progression-free and overall survival [30]. Additionally, age older than 55 years was associated with poorer overall survival, which may be reinforced by the lack of a non-relapse cohort in this group along with GVHD [26]. Furthermore, patients with higher pre-allo-HCT treatment exposure and those with initially poorer treatment response resulted in worse relapse outcomes post-allo-HCT [26]. As with any high-risk treatment, the identification of beneficial and hazardous features pre-allo-HCT treatment should inform clinical decisions as a whole, rather than excluding the potentially curative treatment [26].

Genetically defined high-risk MM in a subset of young patients with 17p deletions may experience limited remissions despite consistent therapeutic intervention [27]. Several studies discuss high-risk cytogenetics and the prognostic impact of allo-SCT on long-term survival [27]. The study by Eisfeld et al. strengthens that delays in transplantation for patients with refractory disease results in poorer outcomes, while a careful selection of high-risk patients who are in earlier phases of disease benefit from allo-SCT while monitoring polyclonal immunoglobulins at least one year post-allo-SCT may identify mortality or relapse threats [27]. Nevertheless, identification of high-risk factors pre-transplant and careful monitoring post-transplant are essential to therapeutic success and full utilization of the potentially curative nature of allo-SCT. The use of allo-SCT in the future treatment of MM is hopeful in combination with existing and yet to be defined treatment regimens; however, further data are needed to clearly define its treatment potential. Future work should focus on stratifying pre-transplant factors with the use of successful therapy combinations such as drug-treatment regimens and allo-auto transplant protocols.

Limitations

Allo-SCT seems to have a promising future in the continued treatment of MM; however, some limitations are foreseeable due to the chronic nature of the disease and long-term follow-up necessary to provide insight into treatment efficacy. In this study, there are limitations in that we focused on PubMed and ScienceDirect indexed articles published in the English language, and studies before 2016 were not included. Our review focused on original research in the form of either observational studies or RCT; however, further insight into a conglomeration of research could be gained by assessing other review articles as well.

## Conclusions

In this systematic review, we aimed to evaluate the current literature on the use of allo-SCT in MM patients and under what circumstances this approach is feasible, preferred, and cautionary. Our review assessed studies that discussed the treatment of MM patients with allo-SCT or any outcomes in MM patients related to allo-SCT. We discussed that while allo-SCT may benefit high-risk patients, successful procedures may incorporate a tandem auto-SCT approach. Moreover, pharmacologic contributions are especially appreciated in allo-SCT due to an observed synergy in the modulation of the immunologic microenvironment. Tailored patient selection is a necessary consideration in projected clinical decisions. By evaluating pre-transplant factors including high-risk cytogenetics, age, and pre-salvage ISS, an estimated success rate can improve expected outcomes in allo-SCT-treated MM patients. The future of allo-SCT in MM treatment may benefit from large-scale studies. Given the chronic nature of MM and long-term follow-up necessary to achieve long-term survival data, subsequent reports should keep in mind the advancements in drug therapy and continue to revise and update the data pertaining to achievable results. Specific recommendations based on peri-transplant factors should also be further clarified in successive research. 
